# In vitro and in vivo evaluation of 2-aminoalkanol and 1,2-alkanediamine derivatives against *Strongyloides venezuelensis*

**DOI:** 10.1186/s13071-016-1648-5

**Published:** 2016-06-28

**Authors:** Ana L. Legarda-Ceballos, Julio López-Abán, Esther del Olmo, Ricardo Escarcena, Luis A. Bustos, Jose Rojas-Caraballo, Belén Vicente, Pedro Fernández-Soto, Arturo San Feliciano, Antonio Muro

**Affiliations:** Parasite and Molecular Immunology Laboratory, Tropical Disease Research Centre, University of Salamanca (IBSAL-CIETUS), Avda. Licenciado Méndez Nieto s/n, 37007 Salamanca, Spain; Department of Pharmaceutical Chemistry, Faculty of Pharmacy (IBSAL-CIETUS), University of Salamanca, 37007 Salamanca, Spain; Present Address: Departamento de Ciencias Farmacéuticas, Facultad de Ciencias, Universidad Católica del Norte, Antofagasta, Chile; Present Address: Centro de Investigación en Salud para el Trópico (CIST), Carretera Troncal del Caribe, Sector Mamatoco, Santa Marta, Magdalena Colombia; Present Address: Facultad de Medicina, Universidad Cooperativa de Colombia, Carretera Troncal del Caribe, Sector Mamatoco, Santa Marta, Magdalena Colombia

**Keywords:** Strongyloidiasis, Treatment, Alkaneaminoalcohol, Alkanediamine, Anthelmintics

## Abstract

**Background:**

Strongyloidiasis is a parasitic disease widely present in tropical and subtropical areas. *Strongyloides stercoralis* represents the main species that infects human beings. Ivermectin is the current drug of choice; however, issues related with treatment failure in patients with diabetes or infected with T-lymphotropic virus-1 make the identification of new molecules for alternative treatment a priority. In the present study, the activity of sphingosine-related aminoalcohol and diamine were evaluated against *Strongyloides venezuelensis* third-stage larva (L3) cultures and experimental infections in mice.

**Methods:**

The efficacy of each compound against L3 was assessed using both XTT (2,3-bis-(2-methoxy-4-nitro-5-sulfophenyl)-2H-tetrazolium-5-carboxanilide) assay and microscopic observation with concentrations ranging from 1 to 350 μM. Cytotoxicity was evaluated using J774.2 macrophage cell line and XTT assay. Lethal concentration 50 (LC_50_), selectivity index (SI) and structure-activity relationships were established. The activity compounds **4** (2-(ethylamino) hexadecan-1-ol), **6** (2-(butylamino) hexadecan-1-ol), **17** (*tert-*butyl *N*-(1-aminododecan-2-yl) carbamate) and **18** (*tert*-butyl *N*-(1-aminohexadecan-2-yl) carbamate) were further assessed against experimental *S. venezuelensis* infections in CD1 mice measuring reductions in the numbers of parthenogenetic females and egg passed in faeces. Mice were infected with 3,000 L3 and treated with 20 mg/kg/day for five days.

**Results:**

In the screening study of 15 aminoalcohols [lauryl (*n* = 9); palmityl (*n* = 13); stearyl (*n* = 15) and alcohol derivatives], the presence of a palmitol chain was associated with the highest efficacy against L3 (LC_50_ 31.9–39.1 μM). Alkylation of the 2-amino group with medium size fragments as ethyl or n-butyl showed the best larvicidal activity. The dialkylation did not improve efficacy. Aminoalcohols **4** and **6** showed the highest SI (1.5 and 1.6, respectively). With respect to diamine derivative compounds, a chain size of sixteen carbon atoms (palmitoyl chain, *n* = 13), and the alkylation of the 2-amino group with medium-sized fragments, were associated with the highest lethal activities. The presence of carbamoyl group in diamines **17** and **18** yielded high SI (1.7 and 1.4, respectively). Infected mice treated with aminoalcohol **6** showed reduction in parthenogenetic females (59 %) and eggs in faeces (51 %).

**Conclusions:**

These results support the potentiality of aminoalcohol and diamine sphingosine-related compounds as suitable prototypes for developing new promising drugs against strongyloidiasis.

**Electronic supplementary material:**

The online version of this article (doi:10.1186/s13071-016-1648-5) contains supplementary material, which is available to authorized users.

## Background

Strongyloidiasis is a parasitic disease caused by nematodes of the genus *Strongyloides*, which are widely present in tropical and subtropical areas with climate suitable for the survival of larval stages of the species of this genus. The main species causing the disease in the human beings is *Strongyloides stercoralis* and it is estimated that 30 to 100 million people are infected around the world [1]. Strongyloidiasis is also classified as a Neglected Tropical Disease, according to the World Health Organization [2, 3]. This parasite has a complex life-cycle: larvae penetrate the skin of the host and migrate through the bloodstream to the lungs, where they enter into the alveolar spaces, ascending to the respiratory tree, the trachea, the pharynx and finally migrate back through the oesophagus and stomach into the intestine where they reach maturity [4–6]. Parthenogenetic females inhabit the mucosa of the small intestine where the eggs are laid. The rhabditiform larvae (L1) are eliminated together with faeces and develop over a few days in temperate and humid environment to infective third-stage filariform larvae (L3). Minimally symptomatic chronic infection and cutaneous, respiratory or gastrointestinal signs are observed in patients. *Strongyloides stercoralis* has also the ability to cause systemic disseminated infection and hyperinfection syndrome in immunocompromised humans. In most of these cases the outcome of the disease is fatal [7, 8].

Ivermectin is an effective well-tolerated drug against strongyloidiasis, reaching cure rates of 93.1–96.8 % with one single dose administration [9]. On the other hand, treatment failure has been observed in patients co-infected with *S. stercoralis* and human T-lymphotropic virus-1 (HTLV-1) [10] and concomitancy with diabetes [11]. Albendazole, mebendazole and thiabendazole given in multiple doses are also used for the treatment of strongyloidiasis but their efficacy and tolerability is not as efficient as ivermectin [12, 13]. The concern about decreased efficacy in human nematodosis and the possibility of acquired-resistance in treatments of human nematodes is increasing [14, 15]. Additionally, rare population genotypes have shown encephalopathy when treated against *Loa loa* infections with ivermectin [16] making the discovery of new alternative nematicidal drugs a high priority challenge.

Long chain aminoalcohols and diamine derivatives are sphingosine-related compounds considered as key molecules for designing alternative drugs to the current treatment of infectious diseases. Biocidal activity of these compounds has been reported against bacteria such as *Mycobacterium tuberculosis* [17], protozoans such as *Plasmodium* spp. [18] *Leishmania* spp. [19], *Trypanosoma brucei* [20], *T. cruzi* [21], *Trichomonas vaginalis*, *Giardia lamblia* [22], fungi [23] and helminths such as *Schistosoma mansoni* [24] or the nematode *Caenorhabditis elegans* [25]. Alkylphospholipids are molecules structurally related to long-chain aminoalcohols and diamines with promising anticancer, antiprotozoal and anthelminthic activity. Their activity is exerted through the interaction with cell membranes, activating apoptosis [26].

In the present study, we have synthesized and evaluated the anti-*Strongyloides* activity of two series of sphingosine derivative compounds including aminoalcohol and diamine derivatives. We have studied their effect on cultures of third-stage *S. venezuelensis* larvae, and those compounds showing good activity were selected to assess their efficacy against *S. venezuelensis* in experimental infections in mice. We also studied cytotoxicity and structure-activity relationships of these aminoalchol and diamine derivatives.

## Methods

### Animals and ethics statement

Animal procedures complied with the European Union (Di 2010/63/CE) and the Spanish (L32/2007, L6/2013, RD53/2013) regulations on animal experimentation. The University of Salamanca’s Ethics Committee also approved the procedures that were used in this study (Protocol: 48531). Male Wistar rats weighing 80–120 g from the Animal Experimentation facilities of the University of Salamanca (Registration No. PAE/SA/001) and male Specific Pathogen Free (SPF) CD1 mice (Charles River, Barcelona, Spain) weighing 25–30 g were used for the maintenance of *S. venezuelensis* life-cycle and for in vivo experiments in standard conditions. Size of groups was calculated by power analysis [27] using “size.fdr” package for R and following the 3Rs recommendations [28].

### Maintaining *Strongyloides venezuelensis* life-cycle and parasitological techniques

The *S. venezuelensis* strain from the Department of Parasitology (University of Minas Gerais, Belo Horizonte, Brazil) was maintained at the University of Salamanca (biosecurity protocol No. 15/019) by serial in vivo passages in Wistar rats. The infective third-stage larvae (L3) of *S. venezuelensis* were obtained from 3 to 4 day-old vermiculite cultures of faeces from infected rats using a Baermann apparatus. L3 were decontaminated according to the methodology previously reported by Martins et al. [29]. Freshly obtained L3 were washed six times, for twenty minutes each, with distilled water containing 100 IU/ml penicillin, 0.1 mg/ml streptomycin and 0.8 mg/ml fluconazole. Absence of bacterial contamination was confirmed by culturing larvae from each batch on a Petri dish containing blood agar at 28 °C during 24 h. Wistar rats were infected with 6,000 L3 in 0.5 ml phosphate buffered saline (PBS) using a 23-gauge needle syringe to maintain the life-cycle. To perform faecal egg counts, mice were placed on grids over clean moist absorbent paper and allowed to defecate. Individual faecal samples were collected, conserved in a 10 % *v/v* formalin buffered solution and counted in triplicate under a microscope using McMaster technique and egg per gram were reported. The upper half of the small intestine was removed after euthanasia (pentobarbital 100 mg/kg), minced and placed in a sedimentation cup wrapped by 8 layers of gauze in PBS for 2 h at 37 °C. The parthenogenetic females were then collected from the sediment and their number recorded.

### Drugs and sample preparation

**Fig. 1:**
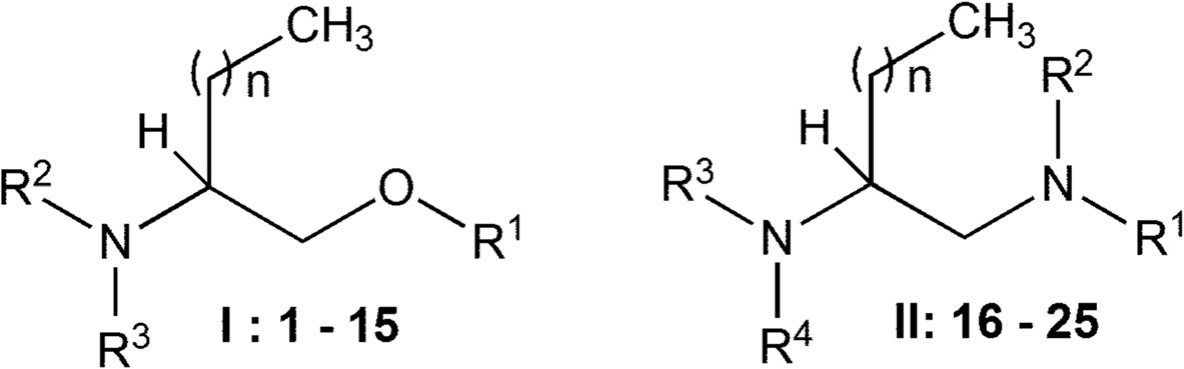
General structures for aminoalcohol derivatives (type I, compounds **1**–**15**) and diamine derivatives (type II, compounds **16**–**25**)

The procedures for the synthesis of 2-aminoalkan-1-ol (type I; compounds 1–15) and alkane 1,2-diamine (type II; compounds 16–25) derivatives, which are structurally related to sphingosine, were previously reported (Fig. 1) [17, 19, 20, 30]. Fifteen aminoalcohol and ten diamine derivatives were synthesized. Compounds were solubilized in dimethyl sulfoxide (DMSO) and appropriate dilutions were made to perform assays. Edelfosine (1-*O*-octadecyl-2-*O*-methyl-*rac*-glycero-3-phosphocholine) was obtained from R. Berchtold (Biochemisches Labor, Bern, Switzerland) and used as reference for in vitro studies. Ivermectin was purchased from Sigma (St Louis, MO, USA) and used as a reference drug for the in vivo treatment of strongyloidiasis.

### Cytotoxicity assessment in mouse macrophage cultures

The J774.2 mouse-derived peritoneal macrophage cell line was used to assess the cytotoxicity of the compounds. Macrophages were grown and serial successive in vitro passages were performed according to the methodology previously described by Rojas-Caraballo et al. [31]. To study the cytotoxicity of each compound, 200 μl of a suspension containing 1 × 10^5^ cells/ml were added onto 96 well flat-bottom microplates, allowed to adhere to the surface of the plate for 2 h at 37 °C, 5 % CO_2_ and then exposed to the compounds for 72 h at the following concentrations: 0.1; 0.5; 3.3; 15.0; 35.0; and 70.0 μM. Each concentration was assayed at least in triplicate in three independent experiments. Cytotoxicity was evaluated based on the colorimetric 2,3-bis-(2-methoxy-4-nitro-5-sulfophenyl)-2*H*-tetrazolium-5-carboxanilide (XTT) assay. After the treatment, 50 μl of XTT solution were added to each individual well and plates were incubated for 24 h at 37 °C. The absorbance of the plate was then measured at 492 nm using an ELISA-plate reader (Anthos Labtec Instruments, Wals, Austria). Results were expressed as the percentage of macrophages remaining alive after each treatment. Untreated and DMSO-treated macrophages were used as controls. Each concentration was assayed in triplicate in three independent experiments and the LC_50_ value for each compound was calculated by sigmoidal regression analysis [32].

### Efficacy and structure-activity study relationship of compounds against third-stage larva cultures

A hundred decontaminated L3 in water were added to 96-well flat bottom microplates. Each compound was added at 1, 10, 35, 70 and 350 μM and then incubated during 72 h at 28 °C in triplicate and in a minimum of three independent experiments. Larval viability was quantified both by using the colorimetric XTT assay as described above and by monitoring larval motility at 24, 48 and 72 h post-treatment. Larval movement was recorded after stimulation with direct natural light for 2 min using an inverted microscope (CK2, Olympus, Tokio, Japan) and a video recorder (AM423 camera and DinoCapture 2.0 software, Dino-Lite digital microscope, Naarden, Holland). Larvae were considered dead when no movement was detected for at least two minutes of detailed examination. As controls, L3 were incubated in water or treated with edelfosine in a range of 1–350 μM. Results were expressed as the concentration of each compound able to inhibit 50 % of total larvae movement (LC_50_), calculated by sigmoidal regression analysis [32]. Selectivity Index (SI) was calculated as the ratio between the LC_50_ value of each compound in macrophage culture and the LC_50_ in L3 cultures to compare the efficacy and toxicity of each compound. Potency relative to edelfosine (P_EDEL_) was calculated as the ratio between edelfosine-LC_50_ and LC_50_ of each compound. Edelfosine, a sphingosine-related compound, was used based on the low toxicity and high in vitro activity against helminths observed in previous experiments in our laboratory [33] The influence of the side-chain size, the substitution of the 2-amino group, and the presence of a free hydroxyl function or a benzyl ether on the activity of each compound were studied in compounds of type I. The influence of the substituents on the 1-amino group and the presence of a *tert*-butoxycarbonyl (Boc) protecting group on the 2-amino group have also been examined in type II compounds.

### Assessment of efficacy against *S. venezuelensis* infection in mice

Those compounds showing the best activity against L3 in the screening test (aminoalcohols **4** and **6**, and diamines **17** and **18**) were tested in *S. venezuelensis* experimental infections of mice. For this purpose, we used a total of 64 male CD1 mice randomly distributed in groups of eight animals. We performed two independent experiments with four groups each: infection control, infected and treated with ivermectin and two groups infected and treated with their respective selected compound. In the first experiment, the aminoalcohols **4** and **6** were tested and in the second experiment the diamines **17** and **18** were tested. All mice were subcutaneously infected with 3,000 L3 of *S. venezuelensis* prior to drug treatment. Ivermectin was orally administered with a single standard dose of 0.2 mg/kg on day 5 post-infection (p.i.). Aminoalcohols and diamines were orally administered with a dose of 20 mg/kg/day at the day of infection and during the next five days to let reaching therapeutical concentration after day 5 p.i. Doses were determined on the basis of the cytotoxicity and previous experience in our laboratory with using sphingosine-related compounds [23, 34]. The number of parasitic females in the gut on day 7 p.i. and the number of eggs released in the faeces during the experiment were quantified to evaluate the efficacy of the treatment.

### Statistical analysis

The results were expressed as the mean and the standard error of the mean (SEM). Normality of data distribution was assessed by the non-parametric Kolmogorov-Smirnov test and the homogeneity of variance was tested by the Barrett test. Significant differences between groups were found using one-way ANOVA followed by Tukey’s honest significance test. All statistical analyses were considered significant at a *P*-value < 0.05. All analyses and graphics were performed with Prism 5 (GraphPad Software, San Diego, CA) for Mac.

## Results

### Efficacy of aminoalcohols and diamines against *S. venezuelensis* L3

Concerning the in vitro activity against *S. venezuelensis* larvae of the aminoalcohol derivatives (compounds **1**–**15** in Table 1), compounds **4** and **6** showed the highest larvicidal activity (LC_50_ = 35.1 ± 0.1 and 31.9 ± 0.5 μM, respectively), that is 1.4 and 1.5 times, respectively, more potent than edelfosine (LC_50_ = 49.6 ± 0.5 μM) but less than ivermectin (LC_50_ = 0.46 ± 0.1 μM) after 72 h of culture. Compounds **4** and **6** had cytotoxicity values of LC_50_ = 52.0 ± 5.3 μM and LC_50_ = 52.0 ± 4.1 μM, respectively, which were also less toxic than edelfosine or ivermectin for macrophages (LC_50_ = 40.7 ± 7.1 and 1.1 ± 0.1 μM, respectively). The SI for edelfosine and ivermectin were 0.8 and 2.4, respectively, while for compounds **4** and **6** the SI were 1.5 and 1.6, respectively, meaning double selectivity (Table 1; Additional file 1: Video S1, Additional file 2: Video S2 and Additional file 3: Video S3 for compound **4** and Additional file 4: Video S4, Additional file 5: Video S5 and Additional file 6: Video S6 for compound **6**). Compounds **14**, **9**, **11** and **1** also elicited good activity with SI values ranging from 0.7 to 0.5 compared to edelfosine activity. Unfortunately all of these were more toxic than edelfosine.

**Table 1:** *In vitro* efficacy and selectivity measured by XTT of alkane aminoalcohol derivatives against *S. venezuelensis* third-stage larvae (L3)

Compound	R^1^	R^2^	R^3^	*n*	Efficacy against L3	P_EDEL_^a^	Cytotoxicity	SI^b^
					LC_50_ (μM)		LC_50_ (μM)	
					Mean ± SEM		Mean ± SEM	
1	H	H	H	9	97.8 ± 10.6	0.5	17.4 ± 0.4	0.2
2	H	H	H	13	348.4 ± 1.5	0.1	nt	–
3	H	H	H	15	239.3 ± 7.9	0.2	nt	–
4	H	H	Et	13	35.1 ± 0.1^c*^	1.4	52.0 ± 5.3	1.5
5	H	H	Bu	9	297.6 ± 5.4	0.1	37.9 ± 1.9	0.1
6	H	H	Bu	13	31.9 ± 0.5^d*^	1.5	52.0 ± 4.1	1.6
7	H	H	Bu	15	293.3 ± 2.6	0.1	10.0 ± 1.5	< 0.1
8	H	H	Hex	9	122.7 ± 6.4	0.4	43.2 ± 0.9	0.4
9	H	H	Hex	13	84.2 ± 4.3	0.5	10.9 ± 1.3	0.1
10	H	H	Hex	15	112.5 ± 5.3	0.4	54.2 ± 0.9	0.5
11	H	Et	Et	13	90.0 ± 5.7	0.5	2.2 ± 2.2	<0.1
12	H	Bu	Bu	9	169.0 ± 9.2	0.2	63.7 ± 1.2	0.4
13	H	Bu	Bu	13	192.1 ± 7.7	0.2	nt	–
14	Bn	H	H	13	67.6 ± 6.3	0.7	30.2 ± 0.3	0.4
15	Bn	H	Bu	13	232.0 ± 4.5	0.2	nt	–
Edelfosine					49.6 ± 0.5	1.0	40.7 ± 7.1	0.8

*Abbreviations: Bn* Benzyl, *Bu* Butyl, *Et* Ethyl, *nt* not tested, *SEM* standard error of the mean, R^1^ substituent on the hydroxyl group; R^2^ and R^3^ substituents on the amine group

^a^Potency relative to edelfosine (P_EDL_) = Compound-LC_50_ against L3 / Edelfosine-LC_50_ against L3 ^b^Selectivity index (SI) = Compound-LC_50_ to macrophages/Compound-LC_50_ against L3

^c^*Significant increase in P_EDEL_ compared to edelfosine (ANOVA *F*_(15, 62)_ = 413.82, *P* < 0.001; HDS *P* = 0.008)

^d^*Significant increase in P_EDEL_ compared to edelfosine (ANOVA *F*_(15, 62)_ = 413.82, *P* < 0.001;^b^HDS *P* = 0.002)

Ten alkane-1,2-diamines were also tested, compounds **16**–**25** (Table 2). Compounds **17**, **18**, **23** and **24** were more potent than edelfosine, with relative potency values of 1.2, 1.2, 1.2 and 1.4, respectively. Compounds **23** and **24** were more toxic than edelfosine, while SIs of compounds **17** and **18** were 1.7 and 1.4, respectively (Table 2: Additional file 7: Video S7, Additional file 8: Video S8 and Additional file 9: Video S9 for compound **17** and Additional file 10: Video S10, Additional file 11: Video S11 and Additional file 12: Video S12 for compound **18**). Compounds **25**, **16**, **20** and **22** have also shown good activity, ranging from 0.7 to 0.5 compared to edelfosine activity. Compound **16** cytotoxicity was similar to edelfosine while the other three were more toxic.

**Table 2:** In vitro efficacy and selectivity measured by XTT of alkane diamines derivatives against *S. venezuelensis* third-stage larvae (L3)

Compound	R^1^	R^2^	R^3^	*n*	Efficacy against L3	P_EDEL_^a^	Cytotoxicity	SI^b^
					LC_50_ (μM)		LC_50_ (μM)	
					Mean ± SEM		Mean ± SEM	
16	H	H	H	13	75.0 ± 4.1	0.6	40.1 ± 0.4	0.5
17	H	H	Boc	9	39.0 ± 2.9^c*^	1.2	66.6 ± 2.2	1.7
18	H	H	Boc	13	39.1 ± 4.7^c*^	1.2	56.2 ± 3.3	1.4
19	H	H	Boc	15	45.7 ± 9.2	1.0	36.8 ± 2.8	0.8
20	H	Et	H	13	95.4 ± 1.5	0.5	10.5 ± 1.4	0.1
21	H	Bu	H	13	148.3 ± 3.3	0.3	15.5 ± 1.2	0.1
22	H	Hex	H	13	83.6 ± 3.1	0.5	5.2 ± 0.5	0.0
23	H	Hex	Boc	13	38.9 ± 4.6^c*^	1.2	4.9 ± 0.8	0.1
24	Et	Et	H	13	33.4 ± 0.7 ^d*^	1.4	13.2 ± 1.7	0.3
25	Hex	Hex	Boc	13	67.8 ± 7.1	0.7	7.6 ± 0.6	0.1
Edelfosine					49.6 ± 0.5	1.0	40.7 ± 7.1	0.8

*Abbreviations: Bn* Benzyl, *Bu* butyl, *Et* ethyl, *Hex* hexyl, *Boc*, *tert*-butoxycarbonyl, *SEM* standard error of the mean; R1 and R2 substituents on the amine at position C-1; R3 substituents on the amine at position C-2

^a^Potency relative to edelfosine (P_EDL_) = Compound-LC_50_ against L3 / Edelfosine-LC_50_ against L3

^b^Selectivity index (SI) = Compound-LC_50_ to macrophages/Compound-LC_50_ against L3

^c*^Significant increase in P_EDEL_ compared to edelfosine ANOVA: *F*_(10, 72)_ = 216.85, *P* < 0.001; HDS *P* = 0.001

^c*^Significant increase in P_EDEL_ compared to edelfosine ANOVA: *F*_(10, 72)_ = 216.85, *P* < 0.001; HDS *P* < 0.001

Compounds, **4**, **6**, **17** and **18**, which reached the highest SI values, seemed to act in a dose- and time-dependent manner, since larval viability was progressively inhibited to completion as dose and time increased. As a consequence of the global consideration of SI values, larval viability measured by XTT and motility examination, the four compounds were selected for evaluation of their in vivo efficacy in infected mice.

### Efficacy of aminoalcohols and diamines against *S. venezuelensis* experimental infections in mice

**Fig. 2:**
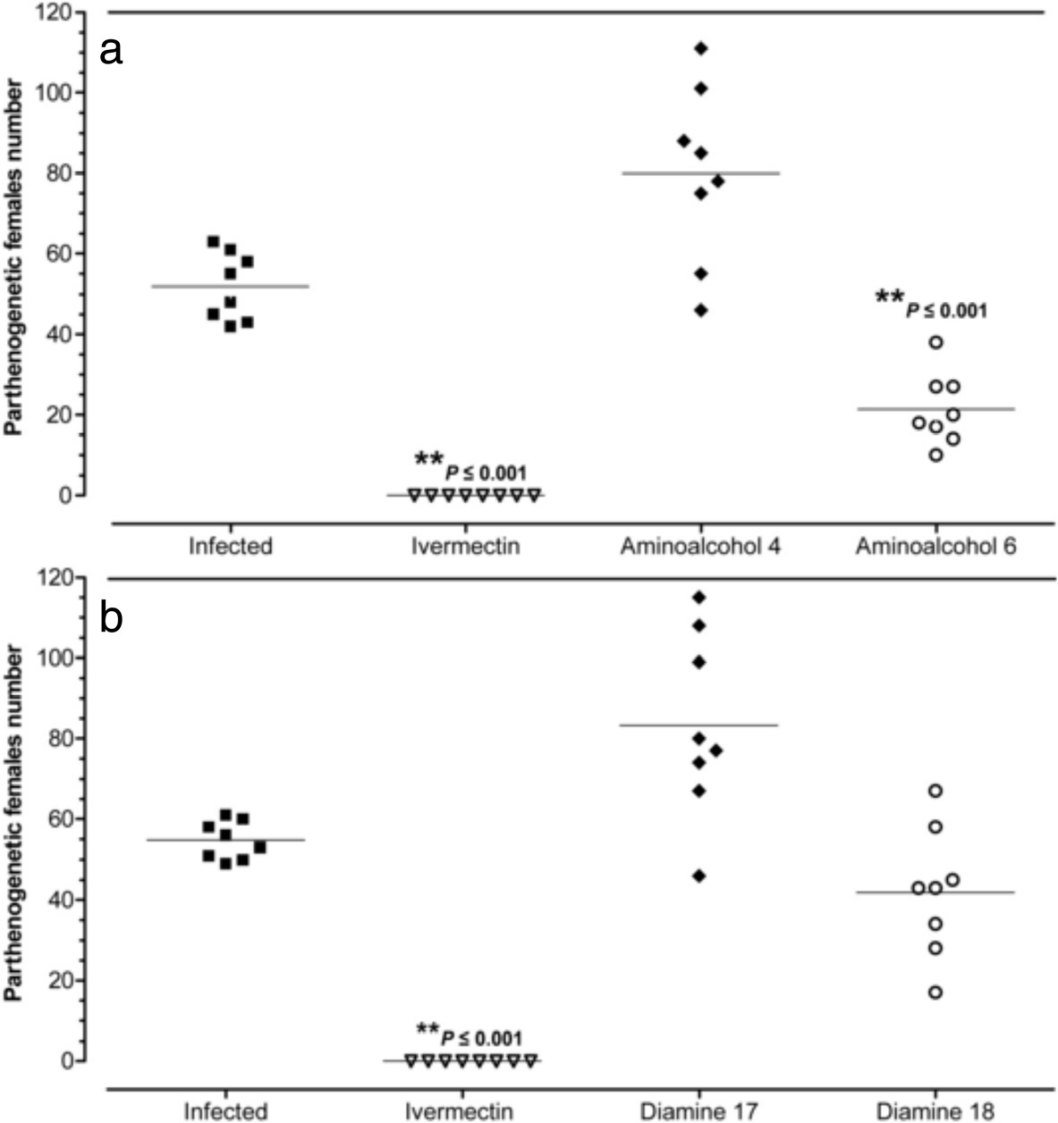
Number of parthenogenetic females on day 7 post-infection. **a** Mice treated with 20 mg/kg of aminoalcohols **4** and **6** (ANOVA *F*_(2, 28)_ = 62.93, *P* < 0.001). **b** Mice treated with 20 mg/kg of diamines **17** and **18** (ANOVA *F*_(3, 28)_ = 48.03, *P* < 0.001). Worms were recovered from the intestine of mice infected with 3,000 *S. venezuelensis* L3 and treated during five days. Each point represents data from individual mice and horizontal bars indicate the means; stars indicate significant reduction of worm recovery compared to infected control

The in vivo anti-*Strongyloides* efficacy of compounds **4**, **6**, **17** and **18** is summarized in Table 3 and Fig. 2. Aminoalcohol **6** induced parthenogenetic female burden reduction of 59 % (Fig. 2a) and significant reductions in egg numbers in faeces on days 6 and 7 p.i. ranging from 35 to 51 %, respectively (Table 3) compared to infection controls. Administration of diamine **18** resulted in reduction in the number of eggs in faeces on day 7 p.i (50 %; Table 3) and moderate but not significant reduction of parthenogenetic females in the gut (25 %; Fig. 2b). Despite aminoalcohol **4** and diamine **17** inducing a moderate reduction of eggs in faeces (45 and 21 %, respectively) they failed to reduce female burdens on day 7 p.i. (Table 3; Fig. 2). During the experiments, all mice used (64/64) remained alive and there was not evidence of any symptoms of severe pain, excessive distress, suffering or an impending death in any of the animals. Daily oral administration of each compound (20 mg/kg/day) and ivermectin (0.2 mg/kg) were well tolerated by animals in all experiments.

**Table 3:** Reduction in egg per gram of faeces (EPGF) in mice infected with 3,000 *S. venezuelensis* L3 after treatment with aminoalcohol derivatives **4** and **6** and diamine derivatives **17** and **18** for five days at a dose of 20 mg/kg, and ivermectin 0.2 mg/kg

Groups	EPGF on day 5		EPGF on day 6		HDS	EPGF on day 7		HDS
	(Mean ± SEM)	Reduction (%)	(Mean ± SEM)	Reduction (%)	*P*	(Mean ± SEM)	Reduction (%)	*P*
Experiment 1								
Infected	2,020 ± 430	–	24,800 ± 5,210	–		12,5150 ± 9,200	–	
Ivermectin	5,240 ± 580	nr	330 ± 110	99^a^	<0.001	0 ± 0	100^a^	< 0.001
Aminoalcohol **4**	3,830 ± 570	nr	20,220 ± 3,080	18	0.274	68,980 ± 12,810	45^a^	< 0.001
Aminoalcohol **6**	4,720 ± 900	nr	16,160 ± 1970	35^a^	0.044	61,680 ± 1,917	51^a^	< 0.001
ANOVA	*F*_(3, 28)_ = 3.28; *P* = 0.051		*F*_(3, 28)_ = 13.42; *P* < 0.001			*F*_(3, 28)_ = 45.31; *P* < 0.001		
Experiment 2								
Infected	3,190 ± 300	–	11,060 ± 1,710	–		82,820 ± 5,364	–	
Ivermectin	2,950 ± 300	8	160 ± 90	99^a^	<0.001	80 ± 50	100^a^	< 0.001
Diamine **17**	2,715 ± 410	15	10,890 ± 1,200	2	0.912	65,520 ± 7,037	21^a^	0.025
Diamine **18**	3,410 ± 520	nr	8,110 ± 430	27	0.060	41,450 ± 5,382	50^a^	< 0.001
ANOVA	*F*_(3, 28)_ = 0.59; *P* = 0.625		*F*_(3, 28)_ = 23.03; *P* < 0.001			*F*_(3, 28)_ = 47.94; *P* < 0.001		

*Abbreviations: HDS* Tukey’s honest significance test, *nr* no reduction, *SEM* standard error of the mean

^a^Significant reduction compared to infected control group

## Discussion

To the best of our knowledge, this study represents the first insight into therapeutic use of alkane aminoalcohols and diamines against a nematode such as *S. venezuelensis*. Synthetic new compounds developed for cancer or organ transplantation are very attractive and untapped resources for the development of new drugs for neglected diseases such as the strongyloidiasis. Long chain-aminoalcohols and diamines are sphingosine-derivatives that have been considered as target molecules for development of new drugs showing cytotoxicity against neoplastic cells [35], and possessing anti-inflammatory properties [34, 36] and ability to kill infectious agents [18, 24, 37]. However, they have never been used against nematodes such as *Strongyloides* spp. These compounds have structural resemblances to anticancer alkylphospholipids with anthelminthic activity against *S. mansoni* and *S. venezuelensis* [38]. Therefore, we used the alkylphospholipid edelfosine as control for in vitro experiments.

We have studied the structure-activity relationships in both type I and type II compounds. We found that aminoalcohols **4** and **6** with a palmitoyl chain (n-hexadecan-1-ol) and the diamines **17** and **18** with chains of lauroyl (n-dodeca-1-ol) and palmitoyl, respectively, showed a more potent activity against *S. venezuelensis* L3, with low cytotoxicity for mammal macrophages and high selectivity indices (SIs) indicating their potential efficacy. Concerning aminoalcohol derivatives (compounds **1**–**15**) the following comparisons of results can be performed. Examination of the substituents of the 2-amino group indicated the preference for a small- (ethyl) to medium- (butyl) sized alkyl group in the secondary amine, as seen in derivatives **4** and **6**, suggesting a decrease in the activity with increase in substituent size increase, compound **9** activity. Interestingly, compound **6** (2-*n*-butylamino-hexadecan-1-ol) was found to be less toxic and had a better SI than the reference drug edelfosine. Compound **6** was also the most potent and selective compound among the aminoalcohols tested. Transformation of the secondary amines **4** and **6** into their respective tertiary analogues **11** and **13** led to a fair reduction of activity. Regarding the 1,2-alkanediamines **16**–**25** (Table 2), the most potent compound against larvae was the diamine **24**, a compound with a primary amine at position C-2 and a tertiary one at C-1; unfortunately, it showed a high cytotoxicity. Compounds **17**, **18** and **23**, the three Boc-protected diamines, showed similar efficacy against L3; the last one was highly toxic, and the other two had SIs of 1.7 and 1.4, respectively. Compounds **17** and **18** only differed in the size of the chain, with respective n-values of 9 and 13; in this case, a small size chain gave less toxicity. In summary, based on the ten alkanediamines tested, it can be concluded that there is a preference for compounds with a Boc-protecting group attached to the 2-amino group in the in vitro activity assays.

Inhibition of the viability of L3 induced by compound **6** increased in a dose- and time-dependent manner, demonstrated using both XTT technique and motility records. Lipophilic diamine and aminoalcohol derivatives containing chains with 13 carbon atoms demonstrate activity against *T. cruzi* trypomastigotes [24], indicating they possess the best range of activity. It seems that the 16-carbon chain length could be involved in the drug’s increased solubility, absorption and harmful action, leading to the dead of the larvae [39]. We observed that only aminoalcohol **6**, containing an alkyl chain with a 16-carbon atom chain and a butyl radical, displayed efficacy against *S. venezuelensis* infection in mice. Although this activity was less effective than ivermectin, the choice drug for strongyloidiasis treatment, our data indicate that oral treatment with aminoalcohol **6** significantly decreases both, the recovered adult parthenogenetic females in the small intestine and the number of eggs per gram of faeces. Aminoalcohol **6** has also demonstrated the highest activity against three *T. cruzi* strains in cultures [21].

Alkylphospholipids, aminoalcohols and diamines have the ability to interact with membrane lipids, allowing their penetration into the parasite where they may disturb cell metabolism and integrity [24]. The pro-apoptotic mechanism was also described in edelfosine [38, 40] and in long chain aminoalcohols [41]. Further studies should be conducted to determine the mechanisms of action. Despite aminoalcohol **6** did not exhibit activity comparable to ivermectin, combinations with current drugs may result in useful synergistic interactions.

## Conclusions

In conclusion, we have reported the strongyloidicidal activity of two series of sphingosine-related compounds, 15 aminoalkanols and 10 alkanediamines, against *S. venezuelensis* using L3 cultures and a strongyloidiasis murine model to search for promising lead compounds that can be optimized to improve their potency and selectivity. This preliminary study introduces aminoalcohol **6** as a suitable prototype for the design of new anti-*Strongyloides* drugs. However, further in vivo studies need to be conducted in order to confirm the outcomes achieved and its utility in therapy against strongyloidiasis and other geohelminthiases.

## Abbreviations

ANOVA, analysis of variance; Boc, *tert*-butoxycarbonyl protecting group; HDS, Tukey’s honest significance test; HTLV-1, Human T-lymphotropic virus-1; L1, first-stage larva or rhabditiform larva; L3 third-stage larva or filariform larva; LC_50_, lethal concentration 50 %; P_EDEL_, Potency relative to edelfosine; SI, selectivity index; XTT, 2,3-bis-(2-methoxy-4-nitro-5-sulfophenyl)-2H-tetrazolium-5-carboxanilide)

## Additional files

Additional file 1: Video S1. Motility in *S. venezuelensis* L3 treated with aminoalcohol **4** at 24 h post treatment under optical microscope. (MP4 6713 kb) Additional file 2: Video S2. Motility in *S. venezuelensis* L3 treated with aminoalcohol **4** at 48 h post treatment under optical microscope. (MP4 6623 kb) Additional file 3: Video S3. Motility in *S. venezuelensis* L3 treated with aminoalcohol **4** at 72 h post treatment under optical microscope. (MP4 6562 kb) Additional file 4: Video S4. Motility in *S. venezuelensis* L3 treated with aminoalcohol **6** at 24 h post treatment under optical microscope. (MP4 6999 kb) Additional file 5: Video S5. Motility in *S. venezuelensis* L3 treated with aminoalcohol 6 at 48 h post treatment under optical microscope. (MP4 6.87 mb) Additional file 6: Video S6. Motility in *S. venezuelensis* L3 treated with aminoalcohol **6** at 72 h post treatment under optical microscope. (MP4 6772 kb) Additional file 7: Video S7. Motility in *S. venezuelensis* L3 treated with diamine **17** at 24 h post treatment under optical microscope. (MP4 7218 kb) Additional file 8: Video S8. Motility in *S. venezuelensis* L3 treated with diamine **17** at 48 h post treatment under optical microscope. (MP4 7202 kb) Additional file 9: Video S9. Motility in *S. venezuelensis* L3 treated with diamine **17** at 72 h post treatment under optical microscope. (MP4 7227 kb) Additional file 10: Video S10. Motility in *S. venezuelensis* L3 treated with diamine **18** at 24 h post treatment under optical microscope. (MP4 7206 kb) Additional file 11: Video S11. Motility in *S. venezuelensis* L3 treated with diamine **18** at 48 h post treatment under optical microscope. (MP4 7297 kb) Additional file 12: Video S12. Motility in *S. venezuelensis* L3 treated with diamine **18** at 72 h post treatment under optical microscope. (MP4 7365 kb)
